# Multidimensional difference analysis in gastric cancer patients between high and low latitude

**DOI:** 10.3389/fgene.2022.944492

**Published:** 2022-07-26

**Authors:** Liqiang Wang, Mengdi Cai, Ying Song, Jing Bai, Wenjing Sun, Jingcui Yu, Shuomeng Du, Jianping Lu, Songbin Fu

**Affiliations:** ^1^ Key Laboratory of Preservation of Human Genetic Resources and Disease Control in China (Harbin Medical University), Ministry of Education, Harbin, China; ^2^ Laboratory of Medical Genetics, Harbin Medical University, Harbin, China; ^3^ Scientific Research Centre, The Second Affiliated Hospital of Harbin Medical University, Harbin, China; ^4^ College of Bioinformatics Science and Technology, Harbin Medical University, Harbin, China

**Keywords:** gastric cancer, genetic variation, metabolic, immune, clinical prognosis

## Abstract

Genetic variation has been shown to affect tumor growth and progression, and the temperature at different latitudes may promote the evolution of genetic variation. Geographical data with latitudinal information is of importance to understand the interplay between genetic variants and environmental influence, such as the temperature, in gastric cancer (GC). In this study, we classified the GC samples from The Cancer Genome Atlas database into two groups based on the latitudinal information of patients and found that GC samples with low-latitude had better clinical outcomes. Further analyses revealed significant differences in other clinical factors such as disease stage and grade between high and low latitudes GC samples. Then, we analyzed the genomic and transcriptomic differences between the two groups. Furthermore, we evaluated the activity score of metabolic pathways and infiltrating immune cells in GC samples with different latitudes using the single-sample gene set enrichment analysis algorithm. These results showed that GC samples at low-latitude had lower tumor mutation burden and subclones as well as higher DNA repair activities. Meanwhile, we found that most immune cells were associated with the prognosis of low-latitude GC patients. At last, we constructed and validated an immune-related prognostic model to evaluate the prognosis of GC samples at different latitudes. This study has provided a further understanding of the geographical contribution to GC at the multiomic level and may benefit the individualized treatment of GC patients at different latitudes.

## Introduction

Gastric cancer (GC) is the most commonly diagnosed cancer in the world and the third leading cause of cancer death (Bray et al., 2018). It is noteworthy that the incidence and mortality of GC vary across geographical regions with different environmental exposure (Voskarides, 2018). Another study showed that cold environmental temperature can increase cancer risk when compared with other factors including alcohol (Sharma et al., 2015). This implicated the involvement of environmental factors in GC development. On the other hand, the mechanism of how the environment may influence the occurrence and progression of gastric cancer remains unclear.

In addition, Walden et al. found that genetic variants play an adaptive role in a changing environment (Walden et al., 2020). However, very few studies exist investigating genetic variants selected for adaptation at regional environmental factors in GC across regions. It is well known that genetic variation is defined as the difference at the DNA level between individuals within the same gene pool. Although genetic variations have been observed in cancer genomics of multiple cancer types, one of the key characteristics of a genetic variant is its geographic distribution (Carrio-Cordo et al., 2020). Studies have found that individuals from different populations carry distinct genetic variants, and low-frequency variation of these shows a significant geographical difference (Genomes Project et al., 2012). Several studies have found that a major cause of tumorigenesis is the accumulation of somatic mutations, which can be influenced by genetic variants and environmental factors (Carrio-Cordo et al., 2020). Pan-cancer analysis of whole genomes from The Cancer Genome Atlas (TCGA) showed that the frequencies of genetic variants appear to be cancer type specific, reflecting various carcinogenesis processes (Consortium, 2020). In addition, genetic variants may be closely related to tumor heterogeneity, which is a natural consequence of genome mutations (Vitale et al., 2021). Tumor heterogeneity can affect the development and progression of cancer by forcing cancer cells into the tumor microenvironment (TME), enabling tumor cells to evade recognition and elimination by the immune system, and thus, it is a critical factor influencing clinical prognosis and response to immunotherapy in GC (Zhang et al., 2021; Sun et al., 2022). Therefore, future studies will need to characterize the geographic distribution of genetic variants of GC samples to understand their contribution to GC development.

In this study, we found significant survival differences between two types of high and low-latitude GC samples from multiple regions. We further analyze somatic mutations on the genome and differential RNA expression on the transcriptome. In addition, we found that there were significant differences in the scores of infiltrating immune cells in TME and tumor-related metabolic pathways in the two types of GC. At last, we constructed an immune-related prognostic model (IRPM) to evaluate the prognosis of GC samples across regions. This study aims to uncover genetic variants in GC across regions through integrative analysis and improve personalized treatment.

## Materials and methods

### Transcriptomic, genomic, and clinical datasets of gastric cancer cohorts

Transcriptional profiles of cancer and normal tissues in GC patients, including mRNA, miRNA, and lncRNA expression profiles, were obtained from stomach adenocarcinoma patients of TCGA (https://portal.gdc.cancer.gov). Two forms of transcriptome datasets were obtained, including expression counts and FPKM standardized data. For cancer or normal samples, RNAs with FPKM expression values of 0 in >70% of samples were removed and the remaining 0 values were imputed with K-nearest neighbors. Then, expression values were log2 transformed for subsequent analysis.

Mutational data of GC patients was also downloaded from the TCGA database. After removing the synonymous variants, we calculated the tumor mutation burden (TMB), which was defined as the number of somatic mutations per megabase of interrogated genomic sequence. The subclone number of each sample, co-occurrence of mutations, and other visualization of mutation profiles were calculated using the R package “maftools” (Mayakonda et al., 2018).

The clinical information of GC patients was obtained from the TCGA database, including survival state, survival time, disease stage, disease grade, therapeutic response, age, gender, and other clinical characteristics.

In addition, the expression profiles and clinical information of two independent validation cohorts were downloaded from the Gene Expression Omnibus (GEO) database, including samples in Houston (GSE26942 and GSE28541) and Seoul (GSE26253). These two cohorts included 257 and 432 samples, respectively.

### Activity score for DNA repair-related GO terms and metabolic pathways

We obtained 46 DNA repair-related GO terms and genes from the Molecular Signatures Database (Liberzon et al., 2015). Based on the FPKM standardized transcriptional data of these genes in the TCGA cohort, we calculated the activity score of each GO term using a single-sample gene set enrichment analysis (ssGSEA) (Rooney et al., 2015).

The metabolic pathways and the related genes were obtained from the KEGG database (Kanehisa et al., 2017). In total, we obtained 77 metabolic pathways. The activity score of each metabolic pathway was also calculated using the ssGSEA algorithm.

### Differential expression of mRNAs, miRNAs, and lncRNAs

Based on the obtained count expression profiles of mRNA, miRNA, and lncRNA (the same number of samples were used to identify differential mRNAs, miRNAs, and lncRNAs, including 74 low-latitude samples, 162 high latitude samples, and 32 normal samples), we identified the differentially expressed RNAs between cancer and normal samples in different latitudes, using the R package “edgeR” (Robinson et al., 2010).

### Infiltration of immune cells in samples

We obtained 28 immune cells and the related 782 marker genes from Charoentong et al. (2017). These marker genes were expressed in specific immune cells. Then, the ssGSEA algorithm was performed to evaluate the infiltrative level of each immune cell in one sample, based on the FPKM standardized expression profile of marker genes.

### Generation of immune score, stromal score, and estimate score

For each patient sample, immune score, stromal score, and estimate score were generated using the R package “estimate” (Yoshihara et al., 2013). A higher score represents a larger ratio of the corresponding component in TME.

### Construction of the driven gene-related metabolic pathway network and the driver gene-related immune cell network

Cancer driver genes in high and low latitudes were identified using the dNdScv method (Martincorena et al., 2017). Then, we identified the regulation relationships between driver genes and metabolic pathways. For each driver gene, the samples were divided into two types with or without the mutations of this given driver gene, and Wilcoxon rank-sum test was performed to evaluate the difference in the activity scores of metabolic pathways between the two sample groups. Those metabolic pathways with *p* values of less than 0.05 were selected to be regarded as regulated by the given driver gene. For a metabolic pathway, if its activity score was significantly higher in mutational samples than that in nonmutational samples, we called it positive regulation. The opposite was called negative regulation. The regulations between driver genes and metabolic pathways were identified respectively in high and low latitudes. Assembling all identified regulation pairs, we generated the driven gene-related metabolic pathway network.

Likewise, we constructed the driver gene-related immune cell network, based on the identified driver gene and activity scores of immune cells.

### Construction of the immune-related prognostic model

We proposed a computational method to establish the IRPM to predict the survival risk for samples in low-latitude, which involved three steps (Bao et al., 2020; Bao et al., 2021; Zhou et al., 2021). First, we evaluated the prognostic effect of each immune cell using a univariate Cox proportional hazards regression model, based on the infiltration profile of immune cells and survival data of samples. The result showed that immune cells mainly have a prognostic effect on samples in low-latitude. Five immune cells with the most significant prognosis for low-latitude samples were selected for subsequent analysis. Second, we screened those top 5% genes with the highest correlation with the infiltration of five prognostic immune cells to be the marker genes, including 35 positive and 12 negative genes. Third, an IRPM score was defined using the *T* statistic of a two-sided *t* test for each low-latitude tumor sample by comparing the expression values of the 35 positively correlated genes with the expression values of the 12 negatively correlated genes (Table 1).

**TABLE 1:** Marker genes used in IRPM model.

	Gene symbols
Positive genes	DOK2, ITGB2, LAPTM5, CD86, CD53, WAS, PLEK, AIF1, MYO1F, CYTH4, ABI3, LSP1, SPI1, CD4, MS4A6A, IL10RA, CYBB, HCST, C1QA, NCKAP1L, C1QB, C1QC, TNFAIP8L2, MNDA, SRGN, SLAMF8, CD84, GMFG, MYO1G, GIMAP4, C3AR1, LILRB1, LST1, HCLS1, HAVCR2
Negative genes	GGH, SQLE, F11R, HMGCS1, TRIP13, MAP7, NUF2, SRP9, PERP, EFNA1, TOP2A, TBCE

The median IRPM score of TCGA low-latitude samples was defined as the cutoff (cutoff = −3.82). An IRPM score > −3.82 represented positive genes were overexpressed, whereas negative genes were underexpressed in this sample. An IRPM score < −3.82 meant the opposite. The five prognostic immune cells were all risk factors, and apparently, the IRPM score was also a risk factor. Using the cutoff, the samples in low-latitude were divided into high- and low-risk groups, with high and low IRPM scores, respectively. The prognostic effect of the IRPM score was validated in the TCGA low-latitude cohort and two GEO low-latitude cohorts (Houston and Seoul cohorts).

### Survival analysis

Kaplan–Meier survival plots and log-rank tests were used to evaluate the survival differences between groups of patients. A univariate Cox proportional hazards regression model was used to evaluate the prognostic significance of metabolic pathways and immune cells. These processes were performed using the R package “survival.”

## Results

### Significant differences in clinical characterizations in samples of high and low latitudes

**FIGURE 1:**
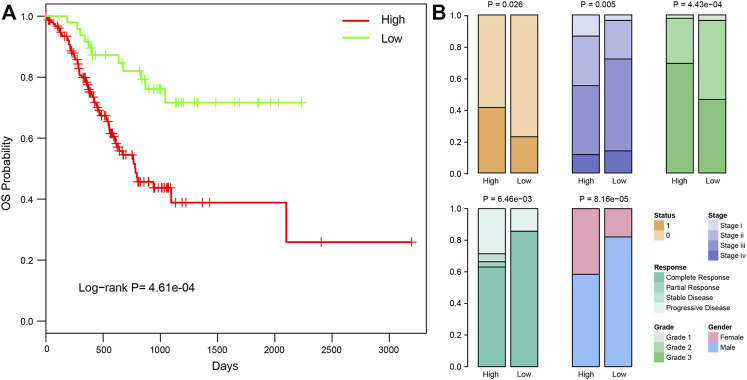
Clinical differences between samples in high and low latitudes. **(A)** Kaplan–Meier curves of overall survival in high and low latitudes of TCGA STAD patients. **(B)** Significantly different clinical factors between samples in high and low latitudes, including survival status, stage, grade, clinical response, and gender. The P and OR values were calculated using Fisher’s exact method.

Studies have shown that average temperature at different latitudes might be significantly associated with cancer death, and cold temperature might contribute to increasing tumorigenesis (Sharma et al., 2015). In this study, we classified the samples into two groups based on the latitude information of patients. Samples at latitudes 0°–45° were defined as low-latitude samples, whereas those at 46°–90° were defined as high latitude samples. Overall survival analysis between these two groups revealed a significantly better clinical outcome for low-latitude samples (log-rank *p* = 4.61e-04, Figure 1A). Comparing other clinical characterizations between the two sample groups, we found various clinical differences (Figure 1B). First, patients in low latitudes showed lower death rates (22.92% vs. 41.40% in low and high latitudes, respectively, Fisher’s test *p* = 0.026). Although the samples in low latitudes had better overall survival, they showed a more advanced disease stage (72.09% vs. 55.23% in low and high latitudes, respectively, Fisher’s test *p* = 0.005). On the other hand, the samples in high latitude were found to be with higher disease grades (69.35% vs. 46.51% samples with grade 3 in high and low latitudes, respectively, Fisher’s test *p* = 4.43e-04) and poor clinical response (63.03% vs. 85.71% complete response in high and low latitudes, respectively, Fisher’s test *p* = 0.006). In addition, we found a higher proportion of male patients in lower latitudes (82.02% vs. 58.42% males in high and low latitudes, respectively, Fisher’s test *p* = 8.16e-05). In summary, these results unraveled the clinical difference between high and low-latitude, which may help to account for the different clinical behaviors of GC patients from different geographical regions.

### Genomic difference between high and low latitudes

**FIGURE 2:**
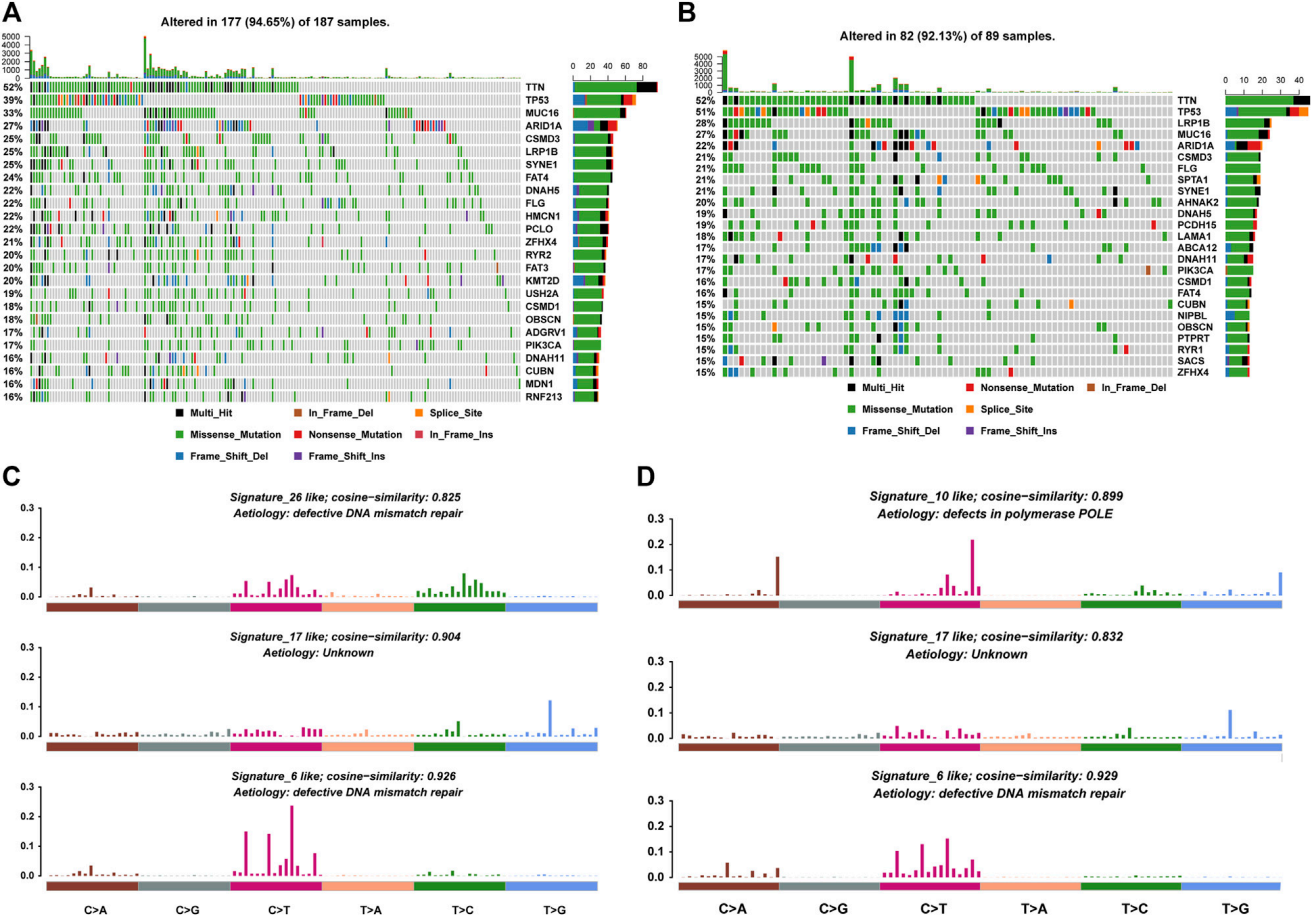
Mutational profiles of high and low latitudes. **(A,B)** Top mutational 25 genes in high and low latitudes. **(C,D)** Mutational signatures in high and low latitudes.

Somatic mutation has been found to be the cause of many cancer types. Therefore, we analyzed the mutational profiles of samples in high and low latitudes, respectively. First, genes with the highest mutation frequency were selected. Figures 2A, B showed the mutation profiles of these genes across high- and low-latitude samples. Comparing the top mutational genes between two groups, we found that 16 out of 25 genes frequently mutated in both sample groups. Meanwhile, we also identified some genes which were frequently mutated in one specific latitude group, including nine genes in high latitudes (HMCN1, PCLO, RYR2, FAT3, KMT2D, USH2A, ADGRV1, MDN1, and RNF213) and nine genes in low latitudes (SPTA1, AHNAK2, PCDH15, LAMA1, ABCA12, NIPBL, PTPRT, RYR1, and SACS). A previous study has found significant differences in the clinical outcome and molecular phenotypes among GC patients when utilizing TP53 and other signaling networks, such as WNT and its related gene RYR1 as biomarkers. This study showed that under the background of TP53 mutations, samples with RYR1 mutation showed significantly better outcomes than those without RYR1 mutations (Park et al., 2016). In our study, we found that RYR1 was mainly mutated in low-latitude samples, which have better clinical outcomes than in high latitude. Another study has proved that the mutations of KMT2D were characterized by increased immune infiltration and could lead to increased DNA damage and mutation burden (Wang et al., 2020). These results were all confirmed in our study in Figure 4A and Figure 7B. In addition, the high frequent mutations of KMT2D in high latitudes and the poor outcome in high latitudes were consistent with their results. Multiple studies have shown that mutations in these genes could influence the overall survival of GC patients (Choi et al., 2015; Chen et al., 2019; Li et al., 2021; Yu et al., 2021). All these results suggest that the different mutational ratios of the same genes and the mutations in specific mutational genes might all be the causes of the difference between the two sample groups. The analysis of mutational signatures can provide important insights into mutational processes associated with cancer development. Therefore, we further recognized the mutational signatures of two sample groups by performing signature enrichment analysis. It is of interest that the mutational signature associated with defective DNA mismatch repair was found to differ across high- and low-latitude samples (Figures 2C, D).

**FIGURE 3:**
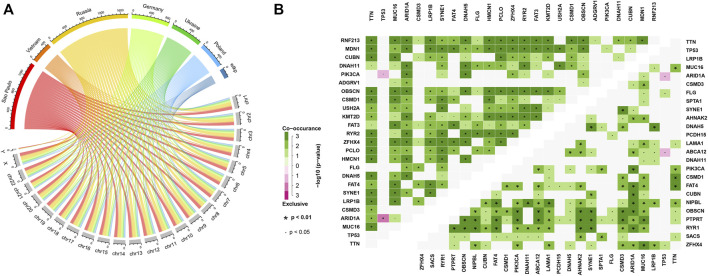
Mutational distribution, co-occurrence, and exclusive analysis. **(A)** Chromosomal mutational distribution of samples in various countries. **(B)** Co-occurrence and exclusive analysis of the top 25 mutational genes in high latitudes (upper left heatmap) and in low latitudes (bottom right heatmap). The significance was colored based on the adjacent color map.

Afterward, we exhibited the mutations of GC patients in each country at the chromosomal level (Figure 3A) and found that mutations were evenly distributed on all chromosomes (detailed list of mutational types of GC samples among various countries in Supplementary Table S1). The mutation frequencies in Russia and Germany were found to be the highest with a mean of 690 and 613 mutations in one sample, respectively. The high level of mutations in these two countries of high latitude might result in their poor clinical outcome. Most top mutational genes were co-occurring in both samples (Figure 3B). However, there were also some exceptions. Mutations in TP53 were exclusively accompanied by PIK3CA in high latitude and ABCA12 in low-latitude, respectively. On the other hand, we found that mutations in TP53 and ARID1A were exclusive in both latitudes. These data indicated that the different mutation profiles may account for the difference between high and low latitudes.

### Samples in high latitude bear higher tumor mutation burden, more subclones as well as lower DNA repair activities

**FIGURE 4:**
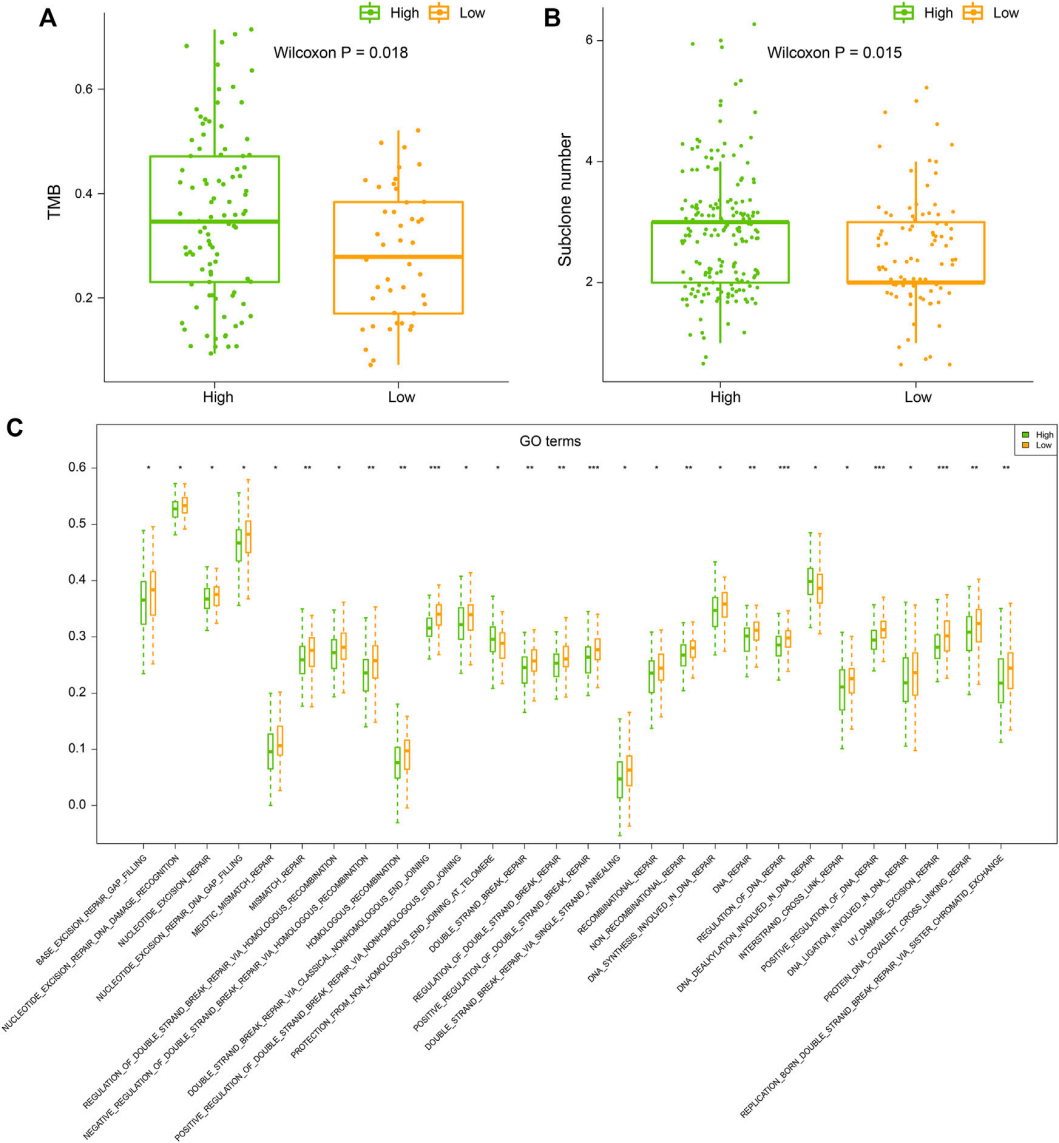
**(A)** Tumor mutation burden (TMB), **(B)** subclone number, and **(C)** ssGSEA score of DNA repair GO terms of samples in high and low latitudes. High and low latitudes were marked green and orange, respectively.

TMB refers to the number of somatic mutations per DNA megabase in tumor cells. It has been found to be the primary driver of antitumor adaptive immune responses and serves as a positive predictive biomarker for immune checkpoint inhibitors (Castle et al., 2012). Meanwhile, subclone is a major manifestation of tumor heterogeneity, which could interfere with the effect of immunotherapy (Vitale et al., 2021). Therefore, we evaluated the TMB and subclone status for each tumor sample. We found that samples in high latitudes have significantly higher TMB and more subclones (Wilcoxon *p* = 0.018 without outline values and *p* = 0.015, Figures 4A, B). Recent studies have illustrated that the genomic instability resulting from deficiency of DNA repair is associated with high TMBs (Picard et al., 2020). Based on the expression profile and DNA repair GO terms, we calculated the activity scores of each DNA repair GO term in each sample. Then, we compared the activity scores of two latitudes. In total, 28 DNA repair GO terms showed significant differences between the two latitudes (Wilcoxon *p* < 0.05). Of note, 27 GO terms showed significantly lower activity scores (Figure 4C). DNA repair was inhibited in high latitude and resulted in the accumulation of somatic mutations (higher TMB). The high level of TMB will accordingly increase the probability of mutations in driver genes and further lead to subclones propagation and high tumor heterogeneity, which will ultimately prompt tumor cells to produce more neoantigens and recruit more immune cells. However, most subclonal tumor cells could escape from the recognition and attack by the immune system instead of being eliminated, which is a major reason for the failure of clinical immunotherapy (Turajlic et al., 2019).

### Transcriptomic difference between high and low latitudes

**FIGURE 5:**
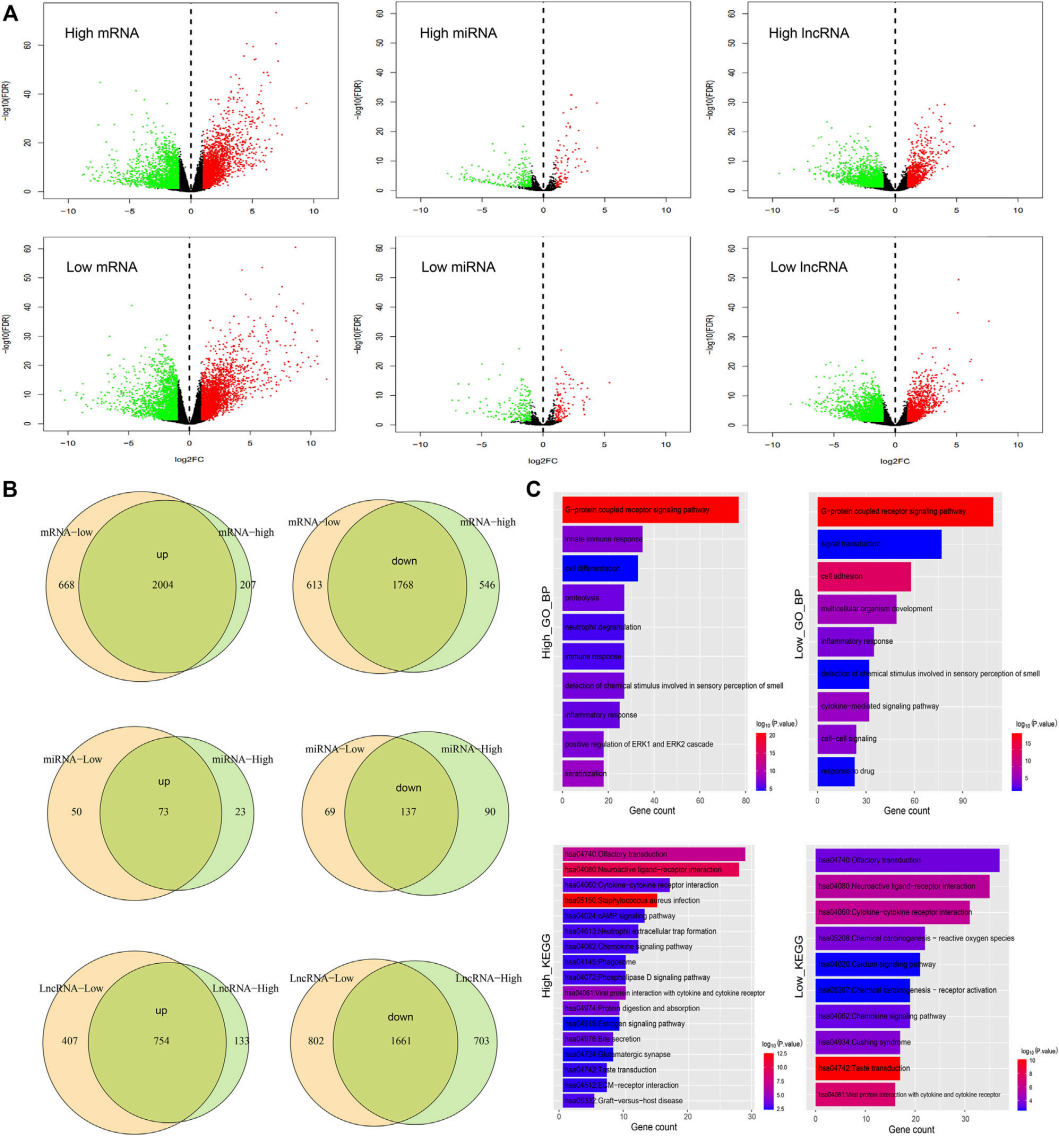
Comparison of samples in high and low latitudes at the transcriptome level. **(A)** Differential expression of mRNAs, miRNAs, and lncRNAs in high- and low-latitude samples. Green and red dots represent the downregulated and upregulated RNAs in cancer samples. **(B)** Overlap of differential upregulated and downregulated mRNAs, miRNAs, and lncRNAs in two sample groups. **(C)** Functional enrichment of mRNAs with differential expression especially in high or low latitude.

It is well known that transcriptomic abnormalities have an important influence on carcinogenesis and cancer progression. Based on the TCGA database, we used the edgeR method and identified the differential expressed mRNAs, miRNAs, and lncRNAs between tumor and normal samples in high and low latitudes (Figure 5A). Results demonstrated that a large portion of differentially expressed RNAs was shared in both groups (Figure 5B), suggesting the consistency at the transcriptomic level between different geographic locations in terms of the same cancer. Meanwhile, hundreds of RNAs were found to be specifically differentially expressed in high or low-latitude, including 668 mRNAs (207 mRNAs), 50 miRNAs (23 miRNAs), and 407 lncRNAs (133 lncRNAs), which were upregulated in low-latitude (high latitude), and 613 mRNAs (546 mRNAs), 69 miRNAs (90 miRNAs), and 802 lncRNAs (703 lncRNAs), which were downregulated in low-latitude (high latitude). We speculate that these specific RNAs might contribute to the different clinical outcomes in samples from different regions. Then, the functional enrichment of these specifically differentially expressed mRNAs was performed in GO biological processes and KEGG pathways (Figure 5C). As a result, we found that genes with specific differential expression at high latitudes were enriched in biological processes of cell differentiation, immune response, and innate immune response. Genes with specifically differential expression at low latitudes tended to be enriched in biological processes of cell adhesion, cell–cell signaling, and response to drug. Furthermore, these genes at two latitudes were collectively enriched in the olfactory transduction pathway, ligand–receptor interaction pathway, cytokine receptor interaction pathway, chemokine signaling pathway, and taste transduction pathway.

### Difference in metabolic activities in high and low latitudes

**FIGURE 6:**
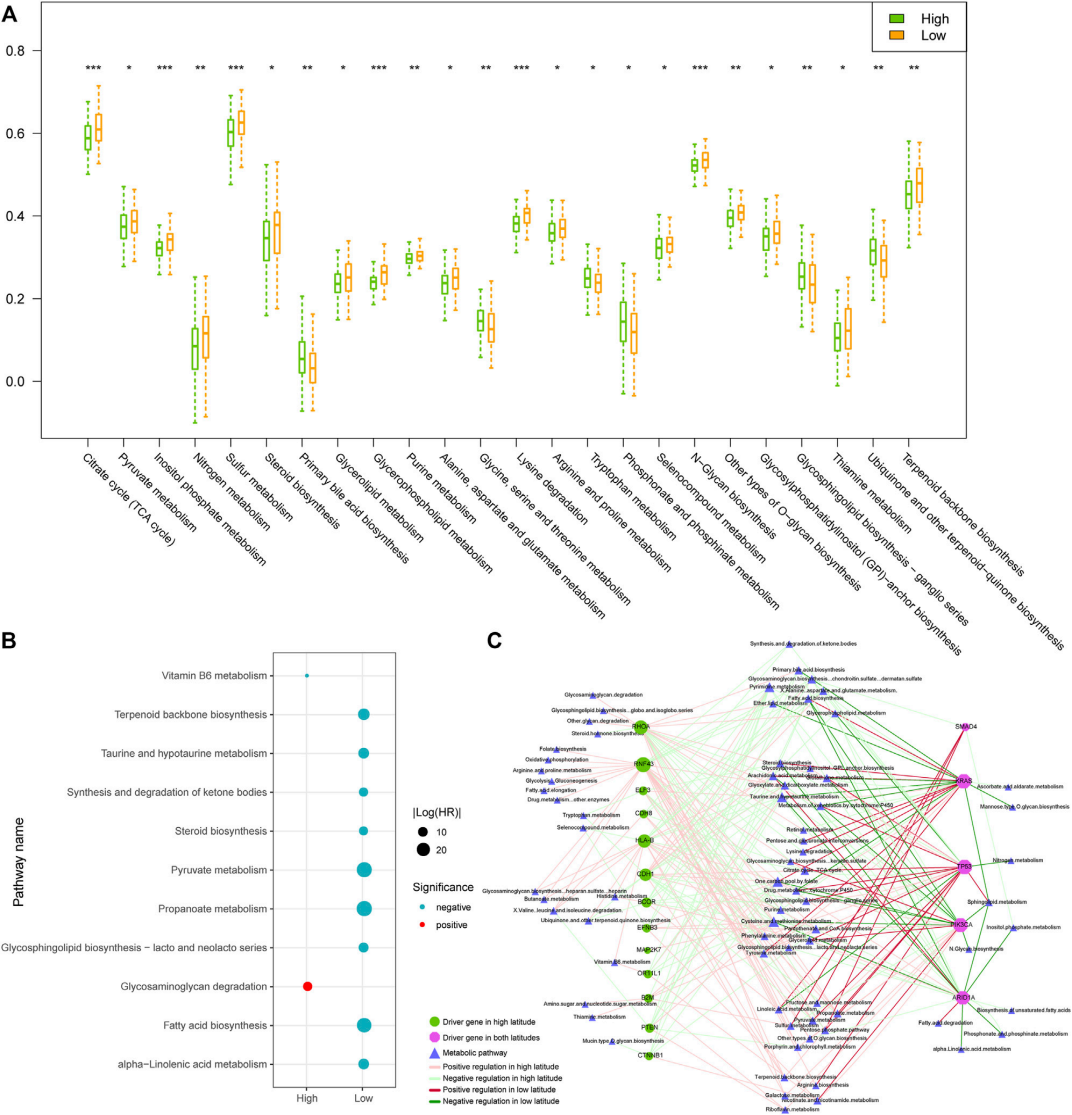
Comparison of samples in high and low latitudes at the metabolic level. **(A)** Metabolic pathways with significantly different ssGSEA activity scores in two sample groups. **(B)** Prognostic metabolic pathways. Blue and red dots represented the protective and risk factors, respectively. **(C)** Driver gene-related metabolic pathway network. The nodes and edges were colored based on the adjacent figure legend.

Numerous studies showed that cancer cells have evolved adaptive metabolic patterns different from normal cells, to meet the needs of tumor growth, maintain a balanced REDOX cell environment, and influence cell communication (Hu et al., 2021; Hu et al., 2022). Cancer cells can also change the tumor microenvironment through reprogramming metabolic patterns and affect the related metabolic activity of tumor cells. Analyzing the metabolic profiles in different regions would help in understanding the metabolic difference and their roles in prognosis. We evaluated the activity score of each metabolic pathway in each tumor sample, using the ssGSEA algorithm based on the obtained metabolic pathways and the expression profile. Then, the activity scores of the metabolic pathways in high and low latitudes were compared. As a result, 24 out of 77 metabolic pathways showed significantly different activity in two latitudes (Wilcoxon *p* < 0.05, Figure 6A). Most of the differential pathways presented lower metabolic activities in high latitude. However, some metabolic pathways presented higher metabolic activities, such as the primary bile acid biosynthesis pathway, the phosphonate and phosphinate metabolism pathway, and the glycosphingolipid biosynthesis–ganglio series pathway.

Furthermore, we used the univariate Cox proportional hazards regression model to evaluate the prognosis of metabolic pathways in tumor samples. Results showed that metabolic pathways have prognostic effects mainly in samples of low-latitude, including nine pathways as protective factors (Figure 6B). The high activities of these metabolic pathways in low-latitude samples positively correlated with the better clinical outcomes of the GC patients. To explore the combination effect of gene mutations and abnormal metabolism on the development of tumors, as described in “Materials and Methods,” we identified the driver genes and their regulated metabolic pathways (Figure 6C). In the network, the metabolic pathways on the left were driven by driver genes only in high latitudes, which is usually enhanced by the mutations in driver genes. Most of the metabolic pathways on the right were weakened by mutations in driver genes. The majority of metabolic pathways were regulated in only one latitude except for several pathways driven by mutational genes in both latitudes. The mutations of driver gene TP53 and KRAS were shared in both latitudes and caused the enhanced activities of tyrosine and pyruvate metabolisms in both latitudes. For Drug metabolism (cytochrome P450 metabolic pathway), KRAS mutations in high latitudes weakened its activity but enhanced its activity in low latitudes. Driver genes usually exerted opposite regulations in two latitudes. On the other hand, mutations in TP53 usually enhanced the metabolic pathways, whereas ARID1A mutations mainly weakened the metabolic pathways. Therefore, the exclusive mutations in TP53 and ARID1A might generate the cellular metabolic programs in tumors.

### Infiltration of immune cells was higher in high latitude than low latitude

**FIGURE 7:**
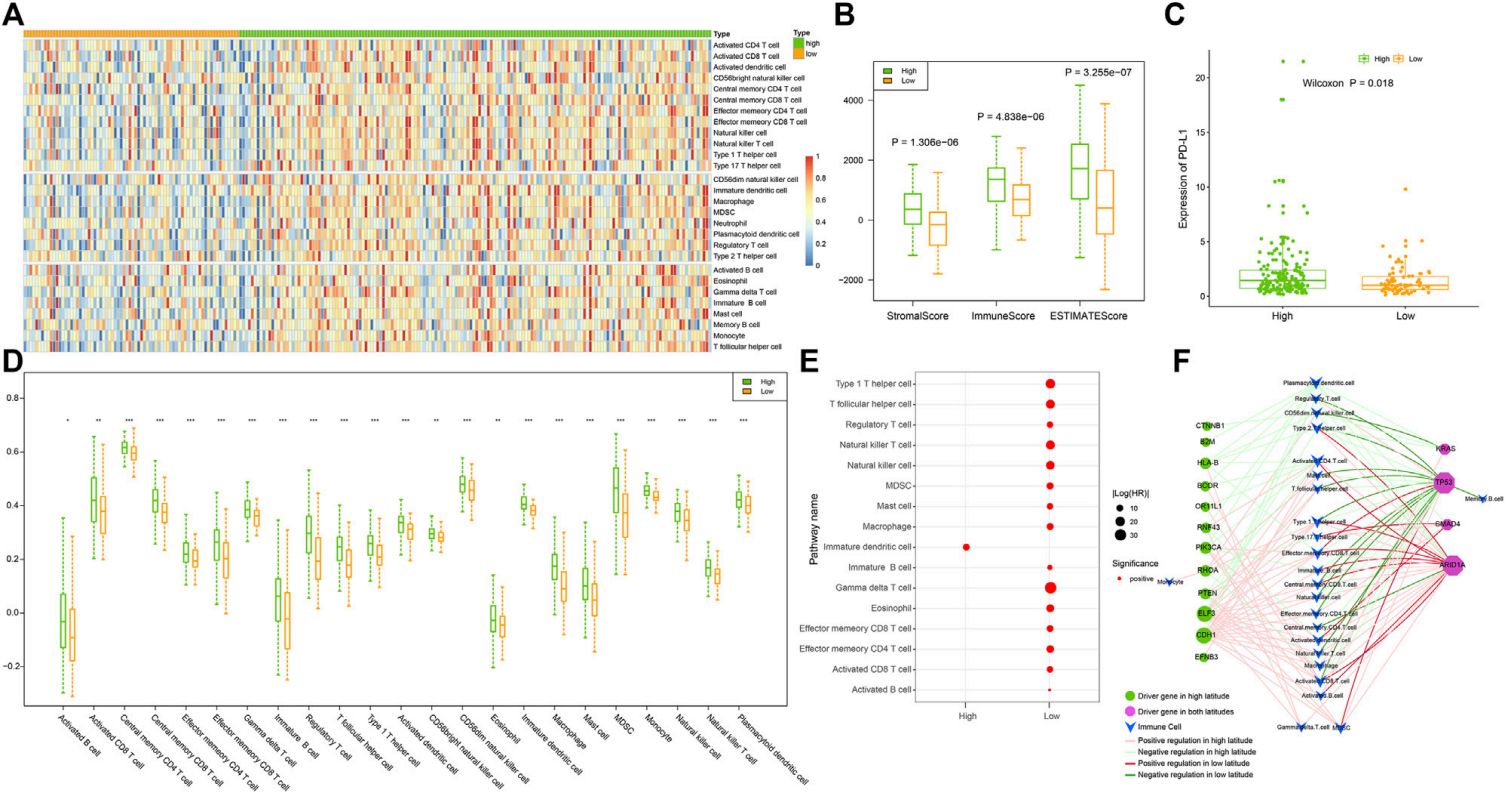
omparison of samples in high and low latitudes at the immune level. **(A)** Infiltration of 28 immune cells in samples. **(B)** Stromal, immune, and ESTIMATE scores of samples evaluated using the “estimate” method. **(C)** Expression of PD-L1 in samples. **(D)** Immune cells with significantly different infiltration between two sample groups; *p* values of less than 0.05, 0.01, and 0.001 were marked with “*”, “**”, and “***,” respectively. **(E)** Prognostic immune cells. **(F)** Driver gene-related immune cell network. The nodes and edges were colored based on the adjacent figure legend.

Infiltrating immune cells are a key element of the tumor microenvironment, and further analysis of the infiltration patterns of these immune cells could help improve the antitumor effect of immunotherapy (Bao et al., 2022). Therefore, we calculated the infiltration of 28 immune cells in GC samples in two latitudes using the ssGSEA algorithm. As shown in Figure 7A, most immune cells presented a higher degree of infiltration in high latitudes, including innate immune cells, such as undifferentiated dendritic cells, neutrophils, and specific immune cells, such as activated CD4 T cells and CD8 T cells. To further clarify the intrinsic biological differences in high and low latitudes, the estimation of stroma and immune cells was used to calculate their scores in malignant tumors by using the ESTIMATE algorithm. The high score estimated in immune and stromal scores represented a large amount of the immune or stromal components in TME. The result showed that the average immune, stromal, and ESTIMATE scores (Figure 7B) were significantly higher in high latitudes than in low latitudes. Recent studies showed that the interaction between PD-L1 on tumor cells and PD-1 on immune cells led to the inactivation of cytotoxic T cells (CTLs such as effector cytotoxic T cells and memory cytotoxic T cells) to evade antitumor immune responses (Batista et al., 2020). Therefore, we further performed a differential analysis of PD-L1 gene expression (Figure 7C) and portrayed a heatmap of immune cells with significant differences by comparing the cell type enrichment level from gene expression data of 28 immune cells in two types. The result indicated that 23 out of 28 immune cells showed significantly different activity in two latitudes (Wilcoxon *p* < 0.05, Figure 7D). Although there were more infiltrating CTLs in high latitude samples, the expression level of the PD-L1 gene was also higher, which might be one of the reasons for the poor immune response.

Then, we utilized a univariate Cox proportional hazards regression model to evaluate the prognosis of immune cells in tumor samples. Data showed that in addition to immature dendritic cells, most immune cells had prognostic effects mainly in low-latitude samples, with 15 immune cells being risk factors (Figure 7E). The lower the activity of these immune cells in low-latitude samples, the better the clinical prognosis of GC patients. We further identified the driver genes and their regulated immune cells (Figure 7F) and found that the immune cells on the left were only driven by driver genes in high latitudes. For instance, HLA-B mutations in the left driver gene could decrease the activity of some immune cells, whereas PTEN, ELF3, and CDH1 mutations could lead to an increase in the activity of some immune cells. In addition, the driver genes on the right were shared by both high and low latitudes. Among them, KRAS and TP53 mutations tend to reduce the activity of immune cells, whereas SMAD4 and ARID1A mutations tend to increase the activity of immune cells. In addition, we found that TP53 and ARID1A regulate immune cells in a mutually exclusive manner.

### Construction and application of the immune-related prognostic model

**FIGURE 8:**
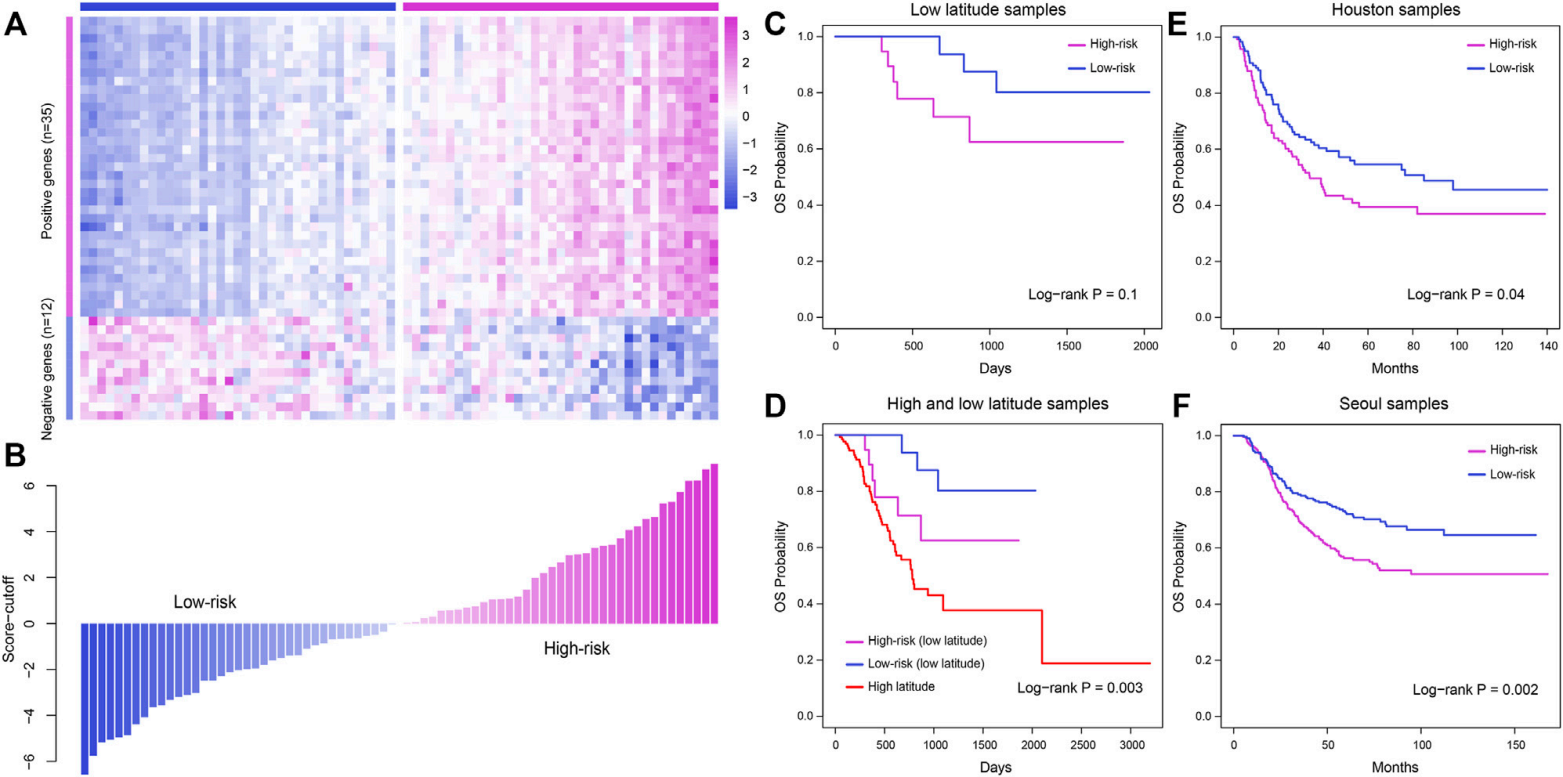
Construction and validation of the IRPM model to predict the clinical outcome for patients in low latitude. **(A)** Expression profile of prognostic immune cell-related marker genes in TCGA low-latitude samples. **(B)** IRPM score of each sample evaluated using the IRPM model. **(C)** Meier survival plot of high- and low-risk samples in low latitudes. **(D)** Kaplan–Meier survival plot of samples in high and low latitudes. **(E–F)** Validation of the prognostic effect of the IRPM model in two GEO cohorts, including Houston samples **(E)** and Seoul samples **(F)**.

Immune cells play a crucial role in tumor development and influence the prognosis of cancer. In our study, most immune cells were found to be related to the prognosis of low-latitude GC patients. In consequence, we constructed an IRPM as described in the Materials and Methods section and further calculated the IRPM score for each sample in low-latitude samples. Kaplan–Meier survival plots were generated and log-rank tests were executed for samples with high and low IRPM scores (with the median score as the cutoff) in low-latitude samples (*n* = 38). As a result, we found that samples with lower IRPM scores had better clinical survival (Figure 8C). The log-rank *p*-value was not significant due to the relatively small number of low-latitude samples, but we found that there was a significant difference in the overall survival probability of high- and low-risk samples by observing the survival curve. In addition, we calculated the predictive effect of IRPM score in high- and low-latitude samples (*n* = 171, log-rank *p* = 0.003, Figure 8D). To further investigate the applicability of the IRPM model and validate its prognostic effect, we collected two independent GEO datasets from low latitudes. Then, we calculated the IRPM score for each sample and further divided the samples into high- and low-risk groups. The results showed that the IRPM model had a significant prognostic effect in two independent GEO datasets (Figures 8E, F).

## Discussion

GC patients from different geographic regions were exposed to multilevel environmental factors, such as geographic factors, life behaviors, and genetic background (Carrio-Cordo et al., 2020). Further study has unraveled the interrelationship of genetic and environmental factors, as environmental factors can influence the process of genetic variation by causing damage to the genome. Therefore, we think that genetic variants could reflect the genetic heterogeneity of GC samples across regions. In this study, we analyze the difference in genome and transcriptome of GC patients from different regions and constructed IRPM, which has been validated in various GEO cohorts, to evaluate the prognosis of GC samples.

Somatic mutations are primarily disease-causing genetic variations in cancer. The most commonly studied mutations are point mutations and fragment mutations, which can be divided into substitutions, deletion, insertions, and inversion according to their mutation types. Genetic alterations showed significant geographical differences (Genomes Project et al., 2012). Some studies indicated that cold temperature could be the possible cause of increased mutation in the genome and might contribute to increasing tumorigenesis (Saini et al., 2017). In our study, we also found that two types of GC samples show differences in mutation type, frequency, mutation signature, tumor heterogeneity, and driver mutation genes. Mutations might have done irreparable damage to DNA and might lead to cell death. DNA repair mechanism plays a crucial role in maintaining genome stability and preventing unfavorable mutations. In this study, we found that most scores of DNA repair-related GO terms of high latitude GC samples were significantly lower than those of low-latitude GC samples. This might be one of the reasons for the difference in TMB levels between the two types of GC samples.

In addition, the number of subclones in high latitude GC samples was significantly higher than in low-latitude GC samples. Recent studies have shown that the number of subclones in a cancer cell has an important effect on tumor heterogeneity (Parikh et al., 2019; Dentro et al., 2021). We found that GC in different regions was highly heterogeneous at the genetic (such as mutations and subclones) level, which has major repercussions on the efficacy of immunotherapy. The high tumor mutational burden favored the infiltration of immune effector cells, mainly because neoantigens were resulting from tumor somatic mutations, which conferred tumor immunogenicity by eliciting antitumor immune responses (Picard et al., 2020). However, antitumor immune responses were negatively correlated with tumor heterogeneity. Although high latitude GC samples had higher TMB levels, they also had more subclone numbers, which might be the reason for the poor immune response in high latitude GC samples.

In summary, we found that the mutation status, in terms of mutation types, mutation number, mutation signature, tumor heterogeneity, and mutation-driving genes, differed dramatically among GC samples from different geographic regions. Further analysis showed that high latitude GC samples had higher scores of infiltrating immune cells but had lower scores of metabolic pathways than low-latitude GC samples. At last, we established an IRPM to predict the clinical outcome for GC samples from different regions. This study has provided a deep understanding of cancer heterogeneity at the genomic and transcriptomic level and facilitated the prediction of the clinical outcome of low-latitude GC patients.

## Data Availability

The original contributions presented in the study are included in the article/supplementary material; further inquiries can be directed to the corresponding authors.

## References

[B1] Bao S. Hu T. Liu J. Su J. Sun J. Ming Y. (2021). Genomic instability-derived plasma extracellular vesicle-microRNA signature as a minimally invasive predictor of risk and unfavorable prognosis in breast cancer. J. Nanobiotechnology 19 (1), 22. 10.1186/s12951-020-00767-3 33436002 PMC7802300

[B2] Bao S. Li K. Yan C. Zhang Z. Qu J. Zhou M. (2022). Deep learning-based advances and applications for single-cell RNA-sequencing data analysis. Brief. Bioinform. 23 (1), bbab473. 10.1093/bib/bbab473 34849562

[B3] Bao S. Zhao H. Yuan J. Fan D. Zhang Z. Su J. (2020). Computational identification of mutator-derived lncRNA signatures of genome instability for improving the clinical outcome of cancers: A case study in breast cancer. Brief. Bioinform. 21 (5), 1742–1755. 10.1093/bib/bbz118 31665214

[B4] Batista A. Rodvold J. J. Xian S. Searles S. C. Lew A. Iwawaki T. (2020). IRE1α regulates macrophage polarization, PD-L1 expression, and tumor survival. PLoS Biol. 18 (6), e3000687. 10.1371/journal.pbio.3000687 32520957 PMC7307794

[B5] Bray F. Ferlay J. Soerjomataram I. Siegel R. L. Torre L. A. Jemal A. (2018). Global cancer statistics 2018: GLOBOCAN estimates of incidence and mortality worldwide for 36 cancers in 185 countries. Ca. Cancer J. Clin. 68 (6), 394–424. 10.3322/caac.21492 30207593

[B6] Carrio-Cordo P. Acheson E. Huang Q. Baudis M. (2020). Geographic assessment of cancer genome profiling studies. Database 2020, baaa009. 10.1093/database/baaa009 32239182 PMC7113738

[B7] Castle J. C. Kreiter S. Diekmann J. Lower M. van de Roemer N. de Graaf J. (2012). Exploiting the mutanome for tumor vaccination. Cancer Res. 72 (5), 1081–1091. 10.1158/0008-5472.CAN-11-3722 22237626

[B8] Charoentong P. Finotello F. Angelova M. Mayer C. Efremova M. Rieder D. (2017). Pan-cancer immunogenomic analyses reveal genotype-immunophenotype relationships and predictors of response to checkpoint blockade. Cell Rep. 18 (1), 248–262. 10.1016/j.celrep.2016.12.019 28052254

[B9] Chen C. Shi C. Huang X. Zheng J. Zhu Z. Li Q. (2019). Molecular profiles and metastasis markers in Chinese patients with gastric carcinoma. Sci. Rep. 9 (1), 13995. 10.1038/s41598-019-50171-7 31570735 PMC6769015

[B10] Choi M. R. An C. H. Yoo N. J. Lee S. H. (2015). Laminin gene LAMB4 is somatically mutated and expressionally altered in gastric and colorectal cancers. APMIS 123 (1), 65–71. 10.1111/apm.12309 25257191

[B11] Consortium I. T. P.-C. A. o. W. G. (2020). Pan-cancer analysis of whole genomes. Nature 578 (7793), 82–93. 10.1038/s41586-020-1969-6 32025007 PMC7025898

[B12] Dentro S. C. Leshchiner I. Haase K. Tarabichi M. Wintersinger J. Deshwar A. G. (2021). Characterizing genetic intra-tumor heterogeneity across 2, 658 human cancer genomes. Cell 184 (8), 2239–2254.e39. 10.1016/j.cell.2021.03.009 33831375 PMC8054914

[B13] Genomes Project C. Abecasis G. R. Auton A. Brooks L. D. DePristo M. A. Durbin R. M. (2012). An integrated map of genetic variation from 1, 092 human genomes. Nature 491 (7422), 56–65. 10.1038/nature11632 23128226 PMC3498066

[B14] Hu W. Jiang C. Kim M. Xiao Y. Richter H. J. Guan D. (2022). Isoform-specific functions of PPARγ in gene regulation and metabolism. Genes Dev. 36 (5-6), 300–312. 10.1101/gad.349232.121 35273075 PMC8973844

[B15] Hu W. Jiang C. Kim M. Yang W. Zhu K. Guan D. (2021). Individual-specific functional epigenomics reveals genetic determinants of adverse metabolic effects of glucocorticoids. Cell Metab. 33 (8), 1592–1609.e7. 10.1016/j.cmet.2021.06.004 34233159 PMC8340270

[B16] Kanehisa M. Furumichi M. Tanabe M. Sato Y. Morishima K. (2017). Kegg: New perspectives on genomes, pathways, diseases and drugs. Nucleic Acids Res. 45 (D1), D353-D361. 10.1093/nar/gkw1092 27899662 PMC5210567

[B17] Li Q. Wu R. Wu F. Chen Q. (2021). KMT2D promotes proliferation of gastric cancer cells: Evidence from ctDNA sequencing. J. Clin. Lab. Anal. 35 (4), e23721. 10.1002/jcla.23721 33793001 PMC8059714

[B18] Liberzon A. Birger C. Thorvaldsdottir H. Ghandi M. Mesirov J. P. Tamayo P. (2015). The Molecular Signatures Database (MSigDB) hallmark gene set collection. Cell Syst. 1 (6), 417–425. 10.1016/j.cels.2015.12.004 26771021 PMC4707969

[B19] Martincorena I. Raine K. M. Gerstung M. Dawson K. J. Haase K. Van Loo P. (2017). Universal patterns of selection in cancer and somatic tissues. Cell 171 (5), 1029–1041 e21. 10.1016/j.cell.2017.09.042 29056346 PMC5720395

[B20] Mayakonda A. Lin D. C. Assenov Y. Plass C. Koeffler H. P. (2018). Maftools: Efficient and comprehensive analysis of somatic variants in cancer. Genome Res. 28 (11), 1747–1756. 10.1101/gr.239244.118 30341162 PMC6211645

[B21] Parikh A. R. Leshchiner I. Elagina L. Goyal L. Levovitz C. Siravegna G. (2019). Liquid versus tissue biopsy for detecting acquired resistance and tumor heterogeneity in gastrointestinal cancers. Nat. Med. 25 (9), 1415–1421. 10.1038/s41591-019-0561-9 31501609 PMC6741444

[B22] Park S. Lee J. Kim Y. H. Park J. Shin J. W. Nam S. (2016). Clinical relevance and molecular phenotypes in gastric cancer, of TP53 mutations and gene expressions, in combination with other gene mutations. Sci. Rep. 6, 34822. 10.1038/srep34822 27708434 PMC5052597

[B23] Picard E. Verschoor C. P. Ma G. W. Pawelec G. (2020). Relationships between immune landscapes, genetic subtypes and responses to immunotherapy in colorectal cancer. Front. Immunol. 11, 369. 10.3389/fimmu.2020.00369 32210966 PMC7068608

[B24] Robinson M. D. McCarthy D. J. Smyth G. K. (2010). edgeR: a Bioconductor package for differential expression analysis of digital gene expression data. Bioinformatics 26 (1), 139–140. 10.1093/bioinformatics/btp616 19910308 PMC2796818

[B25] Rooney M. S. Shukla S. A. Wu C. J. Getz G. Hacohen N. (2015). Molecular and genetic properties of tumors associated with local immune cytolytic activity. Cell 160 (1-2), 48–61. 10.1016/j.cell.2014.12.033 25594174 PMC4856474

[B26] Saini R. Singh A. K. Dhanapal S. Saeed T. H. Hyde G. J. Baskar R. (2017). Brief temperature stress during reproductive stages alters meiotic recombination and somatic mutation rates in the progeny of Arabidopsis. BMC Plant Biol. 17 (1), 103. 10.1186/s12870-017-1051-1 28615006 PMC5471674

[B27] Sharma A. Verma H. K. Joshi S. Panwar M. S. Mandal C. C. (2015). A link between cold environment and cancer. Tumour Biol. 36 (8), 5953–5964. 10.1007/s13277-015-3270-0 25736923

[B28] Sun J. Yan C. Xu D. Zhang Z. Li K. Li X. (2022). Immuno-genomic characterisation of high-grade serous ovarian cancer reveals immune evasion mechanisms and identifies an immunological subtype with a favourable prognosis and improved therapeutic efficacy. Br. J. Cancer 126 (11), 1570–1580. 10.1038/s41416-021-01692-4 35017656 PMC9130248

[B29] Turajlic S. Sottoriva A. Graham T. Swanton C. (2019). Resolving genetic heterogeneity in cancer. Nat. Rev. Genet. 20 (7), 404–416. 10.1038/s41576-019-0114-6 30918367

[B30] Vitale I. Shema E. Loi S. Galluzzi L. (2021). Intratumoral heterogeneity in cancer progression and response to immunotherapy. Nat. Med. 27 (2), 212–224. 10.1038/s41591-021-01233-9 33574607

[B31] Voskarides K. (2018). Combination of 247 genome-wide association studies reveals high cancer risk as a result of evolutionary adaptation. Mol. Biol. Evol. 35 (2), 473–485. 10.1093/molbev/msx305 29220501 PMC5850495

[B32] Walden N. Lucek K. Willi Y. (2020). Lineage-specific adaptation to climate involves flowering time in North American Arabidopsis lyrata. Mol. Ecol. 29 (8), 1436–1451. 10.1111/mec.15338 31850596

[B33] Wang G. Chow R. D. Zhu L. Bai Z. Ye L. Zhang F. (2020). CRISPR-GEMM pooled mutagenic screening identifies KMT2D as a major modulator of immune checkpoint blockade. Cancer Discov. 10 (12), 1912–1933. 10.1158/2159-8290.CD-19-1448 32887696 PMC7710536

[B34] Yoshihara K. Shahmoradgoli M. Martinez E. Vegesna R. Kim H. Torres-Garcia W. (2013). Inferring tumour purity and stromal and immune cell admixture from expression data. Nat. Commun. 4, 2612. 10.1038/ncomms3612 24113773 PMC3826632

[B35] Yu Y. Xie Z. Zhao M. Lian X. (2021). Identification of PIK3CA multigene mutation patterns associated with superior prognosis in stomach cancer. BMC Cancer 21 (1), 368. 10.1186/s12885-021-08115-w 33827485 PMC8028071

[B36] Zhang Z. Bao S. Yan C. Hou P. Zhou M. Sun J. (2021). Computational principles and practice for decoding immune contexture in the tumor microenvironment. Brief. Bioinform. 22 (3), bbaa075. 10.1093/bib/bbaa075 32496512

[B37] Zhou M. Zhang Z. Bao S. Hou P. Yan C. Su J. (2021). Computational recognition of lncRNA signature of tumor-infiltrating B lymphocytes with potential implications in prognosis and immunotherapy of bladder cancer. Brief. Bioinform. 22 (3), bbaa047. 10.1093/bib/bbaa047 32382761

